# Pulsed Laser Deposited Zeolite Coatings on Femtosecond Laser-Nanostructured Steel Meshes for Durable Superhydrophilic/Oleophobic Functionalities

**DOI:** 10.3389/fchem.2021.792641

**Published:** 2021-12-03

**Authors:** Shahbaz Ahmad, M. Egilmez, M. Iqbal, T. Ibrahim, M. Khamis, Ali S. Alnaser

**Affiliations:** ^1^ Department of Physics, American University of Sharjah, Sharjah, United Arab Emirates; ^2^ Department of Chemical Engineering, American University of Sharjah, Sharjah, United Arab Emirates; ^3^ Department of Biology, Chemistry, and Environmental Sciences, American University of Sharjah, Sharjah, United Arab Emirates

**Keywords:** femtosecond laser, nanostructuring, pulsed laser deposition, zeolite coating, oil–water separation

## Abstract

Ultrafast laser structuring has proven to alter the wettability performance of surfaces drastically due to controlled modification of the surface roughness and energy. Surface alteration can be achieved also by coating the surfaces with functional materials with enhanced durability. On this line, robust and tunable surface wettability performance can be achieved by the synergic effects of ultrafast laser structuring and coating. In this work, femtosecond laser-structured stainless steel (SS-100) meshes were used to host the growth of NaAlSi_2_O_6_–H_2_O zeolite films. Contact angle measurements were carried on pristine SS-100 meshes, zeolite-coated SS-100 meshes, laser-structured SS-100 meshes, and zeolite-coated laser-structured SS-100 meshes. Enhanced hydrophilic behavior was observed in the zeolite-coated SS-100 meshes (contact angle 72°) and in laser-structured SS-100 meshes (contact angle 41°). On the other hand, superior durable hydrophilic behavior was observed for the zeolite-coated laser-structured SS-100 meshes (contact angle 14°) over an extended period and reusability. In addition, the zeolite-coated laser-structured SS-100 meshes were subjected to oil–water separation tests and revealed augmented effectuation for oil–water separation.

## Introduction

Industrial wastewater from food and chemical processing, oil refining, and metal structuring consists of water contaminated with oil, which needs an efficient oil–water separation (Ranade and Bhandari, 2014). Moreover, oil spills in oceans cause drastic impact on marine life, human health, and the environment. Hence, the development of cost-effective and highly efficient oil–water separation methods for water purification, pollution control, and oil spill recovery is of great interest. Traditional methods of oil separation techniques from oil-polluted wastewater involve heating, skimming, and chemical dispersion (Rasouli et al., 2021). Despite their effectiveness in oil separation, these methods suffer from producing harmful products leading to a reduction in the oil separation efficiency with time (Rasouli et al., 2021). To overcome this shortcoming, various techniques (Pal, 2017) have been proposed in the literature to separate oil from oil-contaminated wastewater. Recently, porous polymeric membrane-based filters have been widely used in the separation of oil–water due to their high separation efficiency and simple operating conditions (Wang et al., 2018; Bolto et al., 2020). Moreover, nanoparticles mixed with different porous polymers, known as composite membranes, have also been widely used due to their effective selectivity, low cost, and ease of manufacturing (Al-anzi et al., 2017; Noamani et al., 2019). However, the major problem with porous membranes, regardless of their synthesis method, is that they suffer from fouling by organic materials, microplastics, proteins, and biofilms, which reduces their filtration/separation efficiency (Tummons et al., 2020; Junaidi et al., 2021). The accumulated foulants are removed from the surface of membranes by cleaning them in place using strong oxidizing agents. These chemicals, however, can damage the membrane structure and thus decrease membrane lifetime (Zhang et al., 2016; Li et al., 2019). Furthermore, the brine generated from backwashing and cleaning results in the generation of highly toxic waste that results in severe contamination of the environment (Katibi et al., 2021). Thus, the use of clean and sustainable organic membranes for oil–water separation is still a standing challenge to be resolved.

Inorganic membranes based on ceramic and carbon materials exhibit higher efficiencies for oil–water separation and are very durable due to their superior chemical and physical properties (Ding and Gao, 2021; Usman et al., 2021). Ceramic-based membranes are synthesized from oxides (e.g., zirconia, silica, and alumina), zeolites, and metal–organic frameworks (MOFs) (He et al., 2019). Carbon-based membranes involve carbon nanotubes (CNTs) and graphene (Al-anzi et al., 2017). Furthermore, ceramic-based membranes exhibit low fouling, high microbial resistance, and high porosity and possess excellent thermal and chemical properties (He et al., 2019). They are mechanically strong and durable and can withstand high pressure in industrial applications (Lee et al., 2015). However, they are heavyweight, expensive, and physically brittle (Siskens, 1996). On the other hand, although carbon-based membranes such as CNTs and graphene possess high surface area, uniform porosity, tunable surface chemistry, chemical and thermal stability, and high charge conduction (Gu et al., 2016), they show low resistance to fouling and uniform pore size distribution (Fard et al., 2018). Recently, it was reported that coating membranes or metal mesh substrate with functional thin-film structures tunes their physical wettability performance (Wang et al., 2018). In this context, the low surface energy property of the coating adds either tuneable superhydrophobic or tunable superoleophobic functionalities to the substrates (Wang et al., 2018).

Zeolite membranes have attracted considerable attention from researchers around the world for their high porosity, hydrophilicity/oleophobicity, and chemical and thermal stability (Barbosa et al., 2020; Anis et al., 2021). They exhibit microporous 3D crystalline solid structures and contain silicon, aluminum, and oxygen in their framework (Carolyn Rulli). Zeolites are considered to be environmentally friendly and thus have replaced phosphates in many chemical detergents, which led to a significant reduction in water pollution (Li et al., 2017). Owing to their excellent absorption properties and thermal stability, they have also been used in catalytic converters (Premkumar and Balaji, 2020). As effective adsorbents, zeolites are employed in water treatment for the removal of harmful organic pollutants and heavy metals (Li et al., 2017). In particular, sodium-based zeolite (NaAlSi_2_O_6_–H_2_O) has been widely used in membrane applications for oil–water separation due to their isometric trapezohedron structure, which exhibits a large surface area and large pore diameter (>0.5 nm). These properties allow the passage of large ions and molecules through its framework (zeolite | Structure, Properties, and Facts | Britannica, 2020; Mahmodi et al., 2020). As a result, sodium zeolite can be used for better molecular sieving properties. It has been shown in the literature that sodium-based zeolite modifications lead to large pore size substrates with contact angles ranging from 156° to 163.7° with efficient water–oil separation exceeding 99% (Cui et al., 2008; Liu et al., 2018; Mahmodi et al., 2020; Anis et al., 2021). Even though the above-reported methods can efficiently separate oil–water mixtures, they involve toxic chemicals in their synthesis. In addition, they lack durability and longevity (Chu et al., 2015).

Nowadays, surface structuring of pristine metals and alloys with ultrafast laser has been proved to induce effective and durable surface wettability properties (Boltaev et al., 2020; Khan et al., 2021a, 2021b; Yalishev et al., 2021). In this regard, metal surface structuring using femtosecond laser technology has newly emerged as a robust, contactless, and mask-less technique that structures surfaces of any materials with very fine resolution (Toyserkani and Rasti, 2015; Amoako, 2019). Femtosecond laser structuring of different materials is used for many applications such as enhancing the surface area of electrodes for hydrogen production (Amoako, 2019), solar cells (Zhang et al., 2015; Imgrunt et al., 2017), dielectrics (Englert et al., 2008), self-cleaning surfaces (Wu et al., 2021), and water filtration (Zhang et al., 2021). Recently, ultrafast laser structuring of metals and alloys surfaces has been employed in oil–water separation (Zhang et al., 2017; Qin et al., 2019). The laser structuring of surfaces for this application is achieved either by drilling micro-holes through the surfaces or by fine structuring of metal meshes (Qin et al., 2019). Compared with wet chemistry techniques, e.g., hydrothermal and sol-gel, femtosecond laser nanostructuring provides robust, stable, and durable superwetting surfaces for oil–water separation (Yin et al., 2017). Sen et al. fabricated a superhydrophobic titanium filter for oil–water separation by drilling micro-holes through a titanium foil (Ye et al., 2016). Zhou et al. prepared copper filters from Cu sheets by drilling micro-holes with a diameter of 200 μm followed by a raster scan approach to create nanostructures on the surface (Zhou et al., 2019). The sheet was further decorated with graphene oxide through the electrophoresis method to create a superhydrophilic/oleophobic surface with an underwater oil contact angle (OCA) of 165° (Zhou et al., 2019). Titanium oxide film was grown on titanium substrate (TiO_2_@Ti) through femtosecond laser ablation where the laser ablation process oxidized the surface of Ti substrate and formed microchannels with a rough TiO_2_ layer, which created highly stable, self-cleaning, and pollutant-free oil–water filters with high separation efficiency (Cao et al., 2019). Yang et al. structured Ti foam (∼1-mm thickness) using a femtosecond laser (Yang et al., 2019). The surface structured Ti foam exhibited superwetting properties and yielded separation efficiency of 99% for emulsified oil–water separation (Yang et al., 2019). In general, structured surfaces of metals generated as a result of laser ablation demonstrate either superhydrophilic or underwater superoleophobic properties, depending on the type of material, ablation environment, and other laser parameters (Schnell et al., 2019). However, these properties decay significantly with time due to weak adhesion, thus needing proper coating techniques that produce better adhesion (Schnell et al., 2019). Although there are some reports in the literature on surface engineering of metals, alloys, and polymer composites with a femtosecond laser that created durable superhydrophilic and underwater superoleophobic surfaces for oil–water separation, the technique is still rudimentary (Alnaser et al., 2019). Hence, it is very desirable to investigate the combined effect of coating and laser nanostructuring on metal mesh filters.

In this work, sodium zeolite (NaAlSi_2_O_6_–H_2_O)-coated filters with superhydrophilic and underwater superoleophobic properties were prepared. The substrate SS-100 mesh was first nanostructured by a femtosecond laser and later coated with zeolite using the pulsed laser deposition (PLD) technique. The wettability nature of these meshes was evaluated by contact angle measurements and used for oil–water separation. The regeneration of the used zeolite-coated laser-structured SS-100 meshes was achieved by calcination and evaluated for wettability, reusability, and stability. The cycle was repeated for 30 days using water–*n*-hexane mixtures, and the results were compared.

## Materials and Methods

### Materials and Method

SS-100 mesh (316 L), NaAlSi_2_O_6_–H_2_O (99.9% purity), and methylene blue dye were purchased from Sigma-Aldrich (St. Louis, MO, USA). Double distilled deionized (DI) water was used throughout the study.

### Instrumentation

**FIGURE 1:**
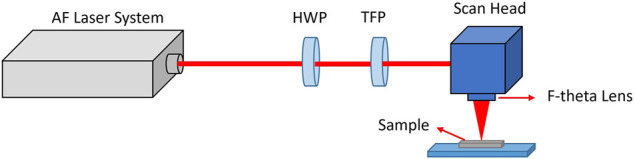
Experimental setup for mesh structuring using a femtosecond laser with half-wave plate (HWP) and a thin-film polarizer (TFP) to control the average power of laser pulses.

An amplified high-powered-based laser system (AFS-UFFL-300-2000-1030-300 from Active Fiber Systems GmbH, Jena, Germany) was employed for the nanostructuring of meshes (Figure 1). F-Theta lens and a raster scanner using the scan head (SH) (FARO tech. Xtreme-20, Faro Technologies, Inc., Lake Mary, FL, USA) was used for laser focusing. Thin-film depositions were performed by Neocera Pioneer 180 PLD system equipped with 248-nm KrF excimer laser (Coherent COMPex Pro 102 F, Coherent, Inc., Santa Clara, CA, USA). Scanning electron microscopy coupled with energy-dispersive X-ray spectroscopy (SEM-EDX) was performed on TESCAN VEGA3 SEM equipped with an EDX spectrometer (Tescan, Brno, Czechia). Phase and crystallographic analyses were performed using X-ray diffraction (XRD) (Malvern Panalytical’s X'Pert³, Malvern, UK). Contact angle measurement was conducted using Drop Shape Analyzer (DSA-100, KRUSS, Matthews, NC, USA).

## Methods

All samples were cleaned with isopropanol followed by DI water and dried in a convection oven before being subjected to laser processing. A femtosecond laser generates a sequence of pulses with a single pulse duration of 40 fs at central wavelength of 1,030 nm, with a single pulse energy of 160 μJ at a repetition rate of 50 kHz. We selected a scanning speed of 100 mm/s with a spacing width of 100 μm between adjacent laser beam paths. The laser beam was initially redirected by mirror systems to a linear attenuator to adjust the laser power, then was focused onto the SS meshes by F-Theta lens, and raster-scanned using the SH, which allowed adjustment of the focal diameter of the laser beam at 60 μm with a laser fluence of 5.6 J/cm^2^.

Sodium zeolite of 2.5-cm diameter × 0.5-cm-thick target for PLD was prepared by hot pressing of fine powder of NaAlSi_2_O_6_–H_2_O. Then thin-film depositions were performed by Neocera (Beltsville, MD, USA) Pioneer 180 PLD system equipped with 248-nm KrF excimer laser. The background pressure in the vacuum chamber was 120 mTorr at a constant flow rate of oxygen. The deposition was performed at 500°C, 21,000 laser pulses, 120-mJ pulse energy, and 7-Hz frequency. This condition produces an 800-nm-thick coating on the mesh surface, which was examined through a SEM. The substrate was stationary while the target was rotated along with rastering during a deposition to circumvent local heating and uniform consumption of the material.

Following the PLD coating, the surface morphology and elemental dispersion analyses of zeolite-coated SS mesh were performed using the SEM-EDX system. Phase and crystallographic analyses on coated meshes were performed using XRD. Wettability characterization of pristine, laser-structured, and coated meshes for air–water contact angle (WCA) and underwater OCAs were performed with Drop Shape Analyzer. The results reported for wettability analysis are the average of three measurements on three different places in the same area of interest. Moreover, for OCA, the untreated surface of meshes was horizontally stuck to glass slides with tape, and the treated surface was dipped in the water for analysis with *n*-hexane oil (2 μl of bubble volume) for each contact angle measurement.

The oil–water separation experiment was performed with 20 ml of water–*n*-hexane mixture (1:1 by volume) and poured on the SS mesh. The two liquids were distinguished by coloring water with methylene blue dye. The total water flux through the mesh and oil intrusion pressure was calculated by Eqs 1 and 2, respectively (Zhang et al., 2020).

$$F=\frac{V}{A\times t}$$ (1)

where *F* is the permeation flux of water, *V* is the volume of the liquid permeated through the mesh area *A* (m^2^), and *t* is the total time of separation.

$$P_{\mathrm{int}}=2\gamma_{\ell 1/2}\frac{Cos\theta }{d}$$ (2)

*P*_int_ is the intrusion pressure (kPa), $\gamma_{\ell 1/2}$ is the oil and water interfacial tension (mN/m), *θ* is the OCA (°), and *d* is the pore size of mesh (m).

## Results and Discussion

### Surface Characterization

**FIGURE 2:**
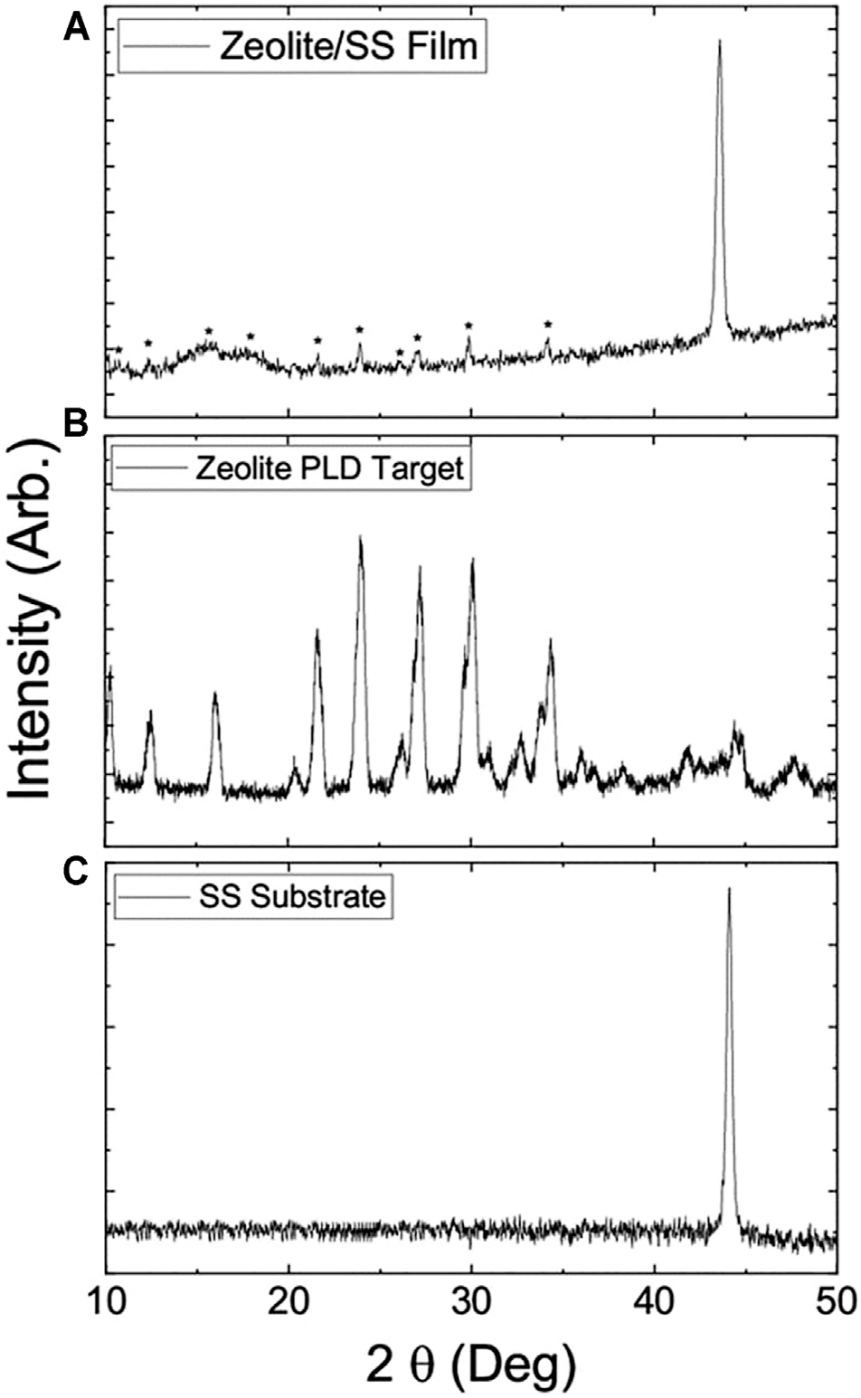
X-ray diffraction spectrum of **(A)** zeolite-coated laser-structured SS meshes (peaks that belong to zeolite are marked with “*”). **(B)** Zeolite target for pulsed laser deposition (PLD). **(C)** SS-100 substrate. The thickness of the film used is 800 nm.

The XRD spectra of zeolite-coated laser-structured SS-100 meshes (Figure 2A), zeolite (Figure 2B), and SS-100 substrate (Figure 2C) were recorded for 800-nm films. Inspection of these spectra reveals that the target zeolite NaAlSi_2_O_6_–H_2_O can be characterized as analcime (JCPDS 41-1478) with a hexagonal crystal structure (Ma et al., 2015; Bisung and Dickin, 2020). On the other hand, the XRD spectra for SS-100 mesh reveal that it can be identified as a polycrystalline material with face-centered cubic (fcc) crystal structure (Pető et al., 2020). Furthermore, the SS-100 mesh spectra show a 2θ peak at 44.5 (111) degrees with additional peaks at 51.1 (200) and 74.9 (220) degrees. These additional peaks could be assigned to the fcc structure (Pető et al., 2020). The spectra of zeolite-coated laser-structured SS-100 meshes (Figure 2A) reveal that the fabricated films were polycrystalline (Ramakrishna et al., 2016). This is due to the thermal treatment during the deposition in which the substrate was kept at 500°C during the deposition. Furthermore, the spectra in Figure 2A reveal that deposition of zeolite was successfully achieved on SS-100 with the appearance of all its peaks marked with *.

**FIGURE 3:**
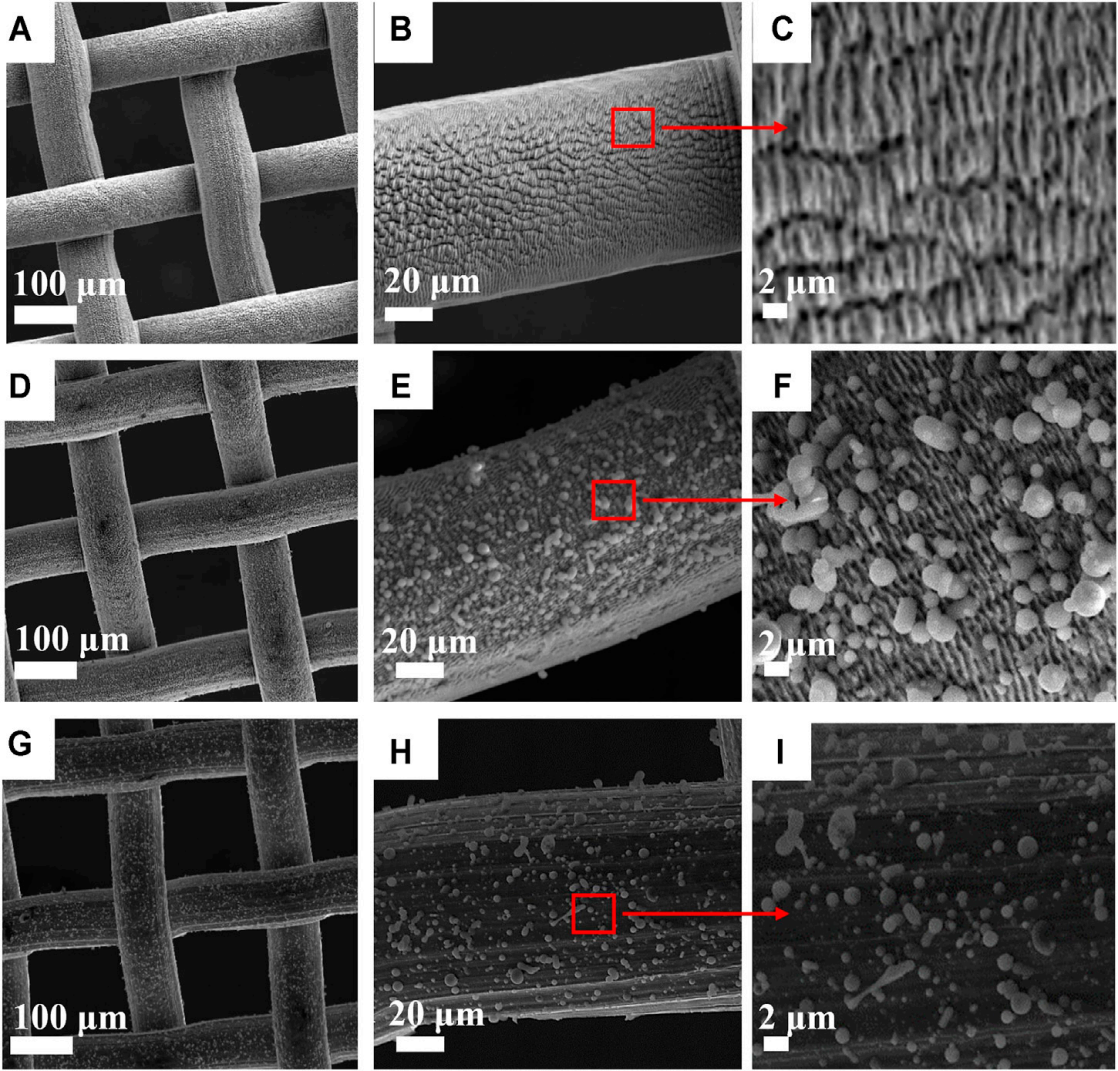
Scanning electron microscopy (SEM) micrograph of SS-100 mesh surfaces. **(A–C)** Uncoated SS-100 mesh surface structured with laser-induced periodic surface structure (LIPSS). **(D–F)** Structured SS-100 mesh with LIPSS after coating with zeolite surface. **(G–I)** Unstructured SS-100 mesh with zeolite-coated surface.

The surface morphology of laser-structured SS-100 mesh was studied through SEM. Figures 3A–C show SEM images of uncoated mesh with laser-induced periodic surface structures (LIPSSs) of 1 μm period. The image is similar to what has been reported earlier by our group using the same LIPSS (Khan et al., 2020). It can be concluded that the induced surface roughness with LIPSS structuring is a key factor for the observed underwater superoleophobic property (Li et al., 2016). The SEM images for the PLD thin film on SS-100 unstructured mesh and LIPSS structured meshes are presented in Figures 3D–I. Inspection of these images reveals that coating with zeolite produced a uniform film with a crystallite-like structure. Furthermore, the zeolite crystallites along with LIPSS induce much higher surface roughness as compared with the uncoated surface (Figure 3C) and unstructured surfaces (Figure 3I). This high surface roughness is believed to be the crucial factor behind the enhancement of surface hydrophilic property (Polini and Yang, 2017).

**FIGURE 4:**
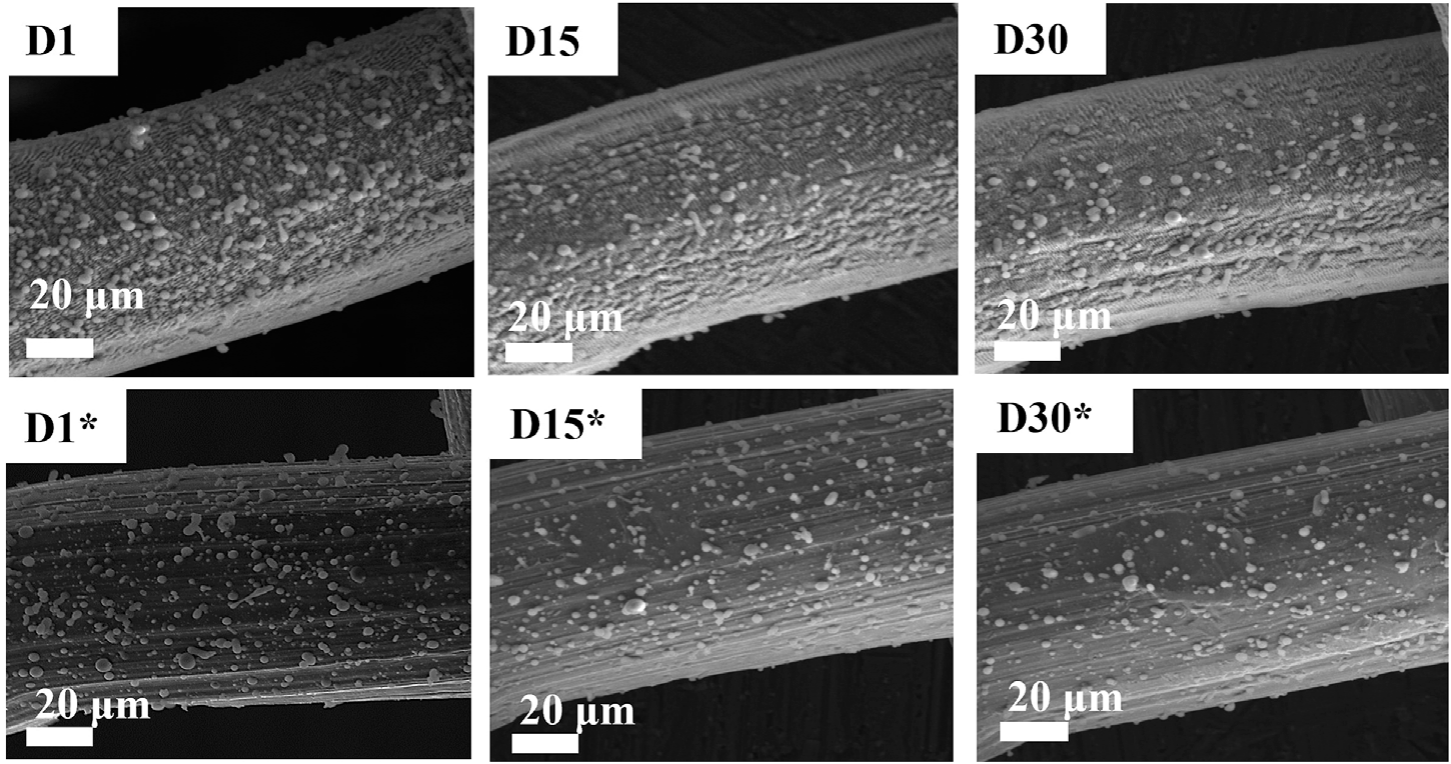
Scanning electron microscopy (SEM) analysis of aging sodium zeolite-coated SS mesh. D1, D15, and D30 represent days, while * represents the unstructured surface.

Figure 4 shows the changes in surface morphology after aging and application in oil–water removal for 30 days. In these experiments, the oil–WCA analysis was done every other day. After each test, the meshes were calcined at 250°C for 20 min to remove hydrocarbons. The reported SEM images (Figure 4) show that the loosely bonded zeolite seeds on the surface of meshes eroded with time during calcination. On the other hand, the LIPSS structured surface with tightly bonded zeolite powder is still there, which was the reason for the permanent surface wettability response.

**FIGURE 5:**
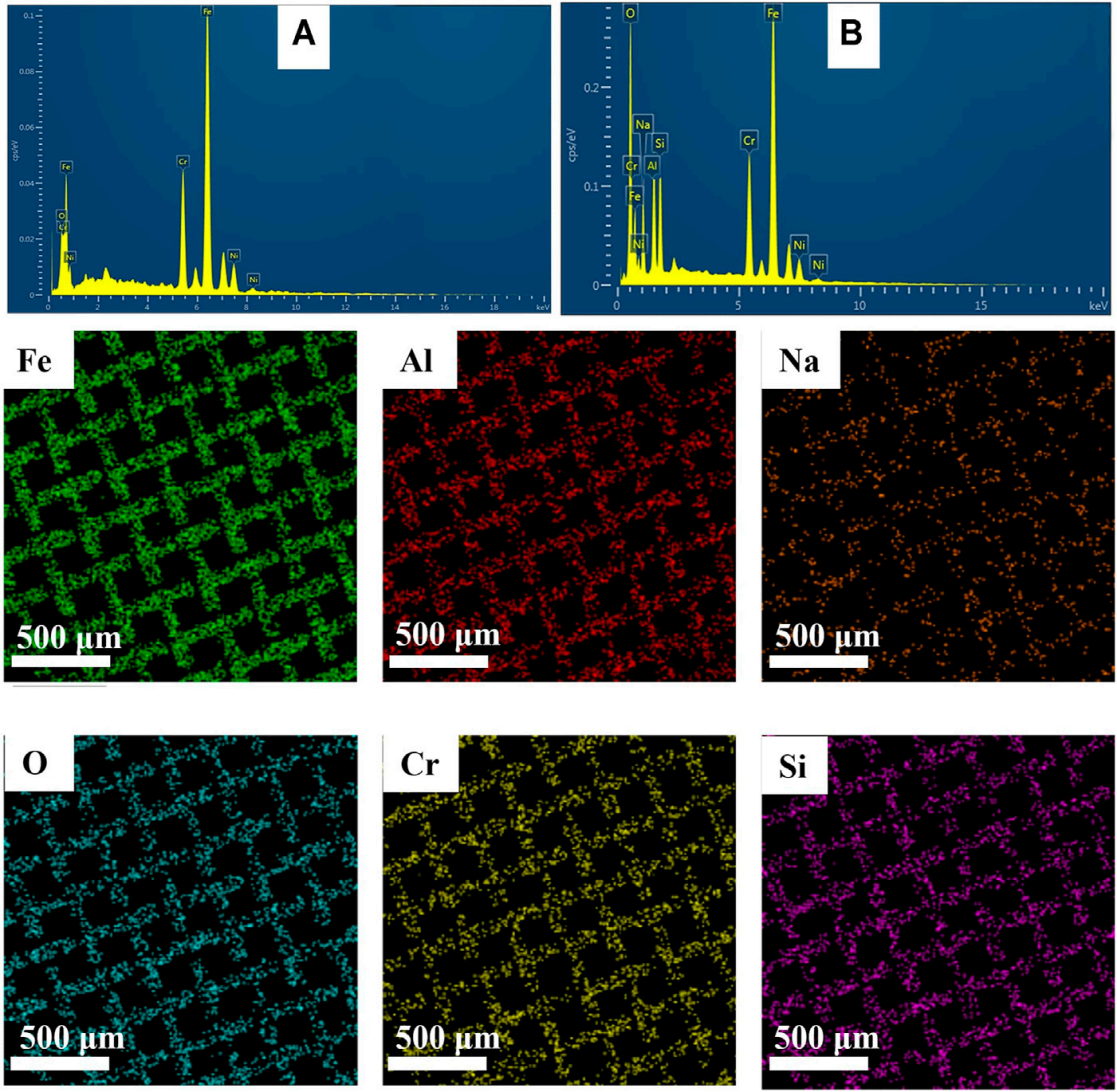
Energy-dispersive X-ray (EDX) spectra and color mapping of pristine and sodium zeolite-coated SS mesh.

For the elemental composition of sodium zeolite coatings, EDX analysis was performed on the pristine and coated meshes. EDX spectra in Figures 5A,B for pristine and coated meshes with zeolite show that Al, Si, and Na were uniformly distributed over the mesh surface at 11%, 7.8%, and 7.3%, respectively, which can be seen in the color mapping below the spectra.

### Surface Hydrophilic/Oleophobic Properties

It was reported earlier that the surface roughness with proper microstructures engineered using a femtosecond laser tends to produce superwetting states with extreme superhydrophilic or superhydrophobic films (Nakae et al., 1998). These unique modified properties of the meshes are of high demand in oil–water separation industries, for they can yield efficient oil removal from contaminated water for environmental remediation. Hence, the development of robust and efficient superhydrophobic or superhydrophilic meshes is attracting major research in this field.

**FIGURE 6:**
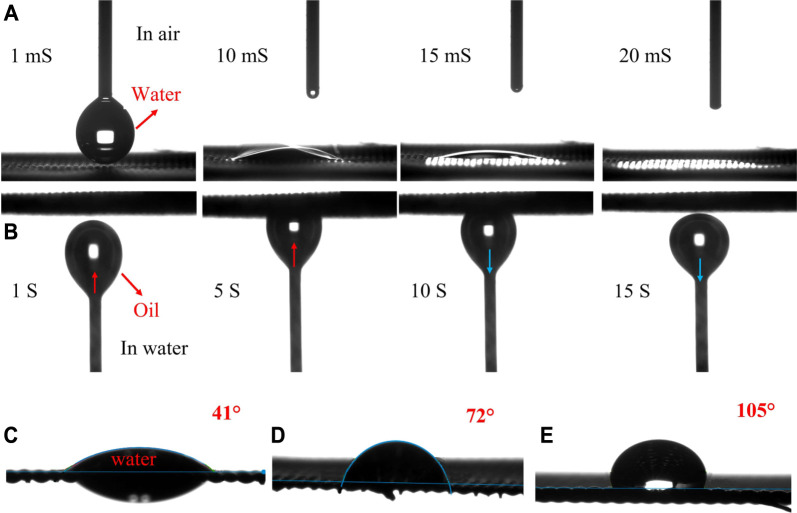
Wettability test of SS meshes. **(A)** Water contact angle (WCA) test for the zeolite-coated laser-structured SS-100 meshes. **(B)** Oil contact angle (OCA) test for the zeolite-coated laser-structured SS-100 meshes. **(C)** WCA for uncoated laser-structured. **(D)** WCA for coated non-laser-structured. **(E)** WCA for pristine SS-100 mesh.

The wettability tests of the laser-structured and coated meshes in the air with water droplets and underwater with oil bubbles were carefully studied by the contact angle method. As shown in Figure 6A (Supplementary Video S1), the water droplet in contact with the zeolite-coated laser-structured SS-100 meshes spread immediately over the surface and permeated completely through the mesh within 20 ms, exhibiting superhydrophilic nature. Furthermore, underwater oil measurements reveal that the zeolite-coated laser-structured SS-100 meshes show superhydrophilic with non-adhesive nature as evident from the deformation of the oil bubble from ellipsoidal to circular shape under slight pressure against the mesh surface (Figure 6B and Supplementary Video S2). It could be deduced that the superhydrophilicity provided the zeolite-coated laser-structured SS-100 meshes with underwater superoleophobicity property (Wu et al., 2018). On the other hand, the uncoated structured, coated laser-unstructured, and pristine meshes are not superhydrophilic and show a WCA of 41°, 72°, and 105°, respectively (shown in Figures 6C–E).

**FIGURE 7:**
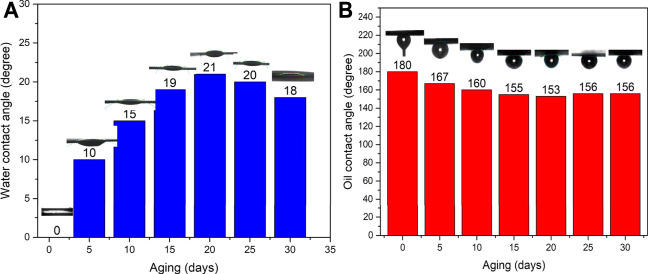
Effect of aging time on the water contact angle (WCA) **(A)** and OCT **(B)** of the zeolite-coated laser-structured SS-100 meshes.

To test the durability of the zeolite-coated laser-structured SS-100 meshes with aging time, WCA and OCA were measured over 30 days and shown in Figures 7A,B. Inspection of this figure reveals that the surface wettability of the zeolite-coated laser-structured SS-100 meshes changed by small angles over 30 days’ period and thus retained their superhydrophilic and underwater superoleophobic property.

### Application in Oil–Water Separation

**FIGURE 8:**
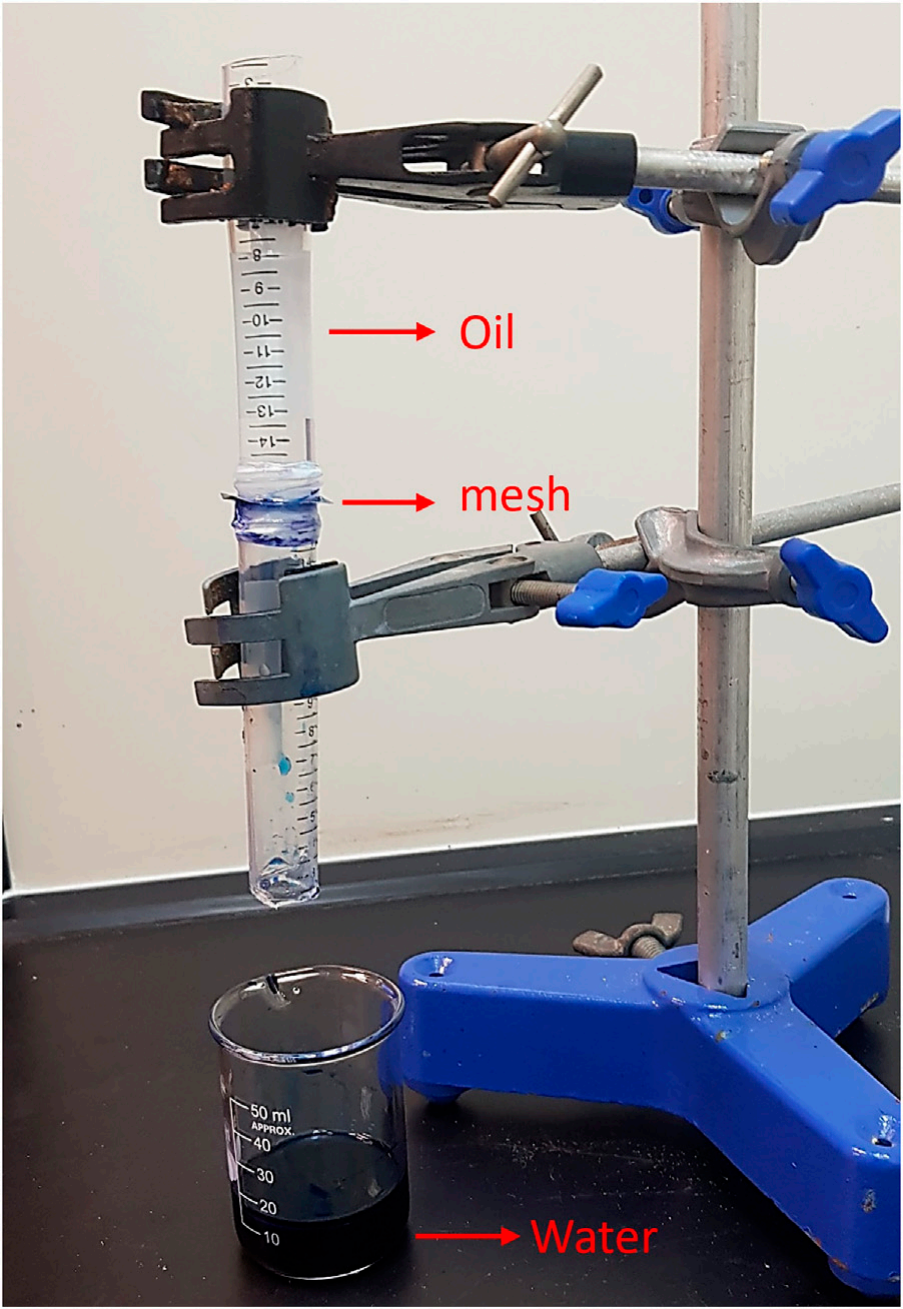
Oil–water separation apparatus using the zeolite-coated laser-structured SS-100 meshes.

**FIGURE 9:**
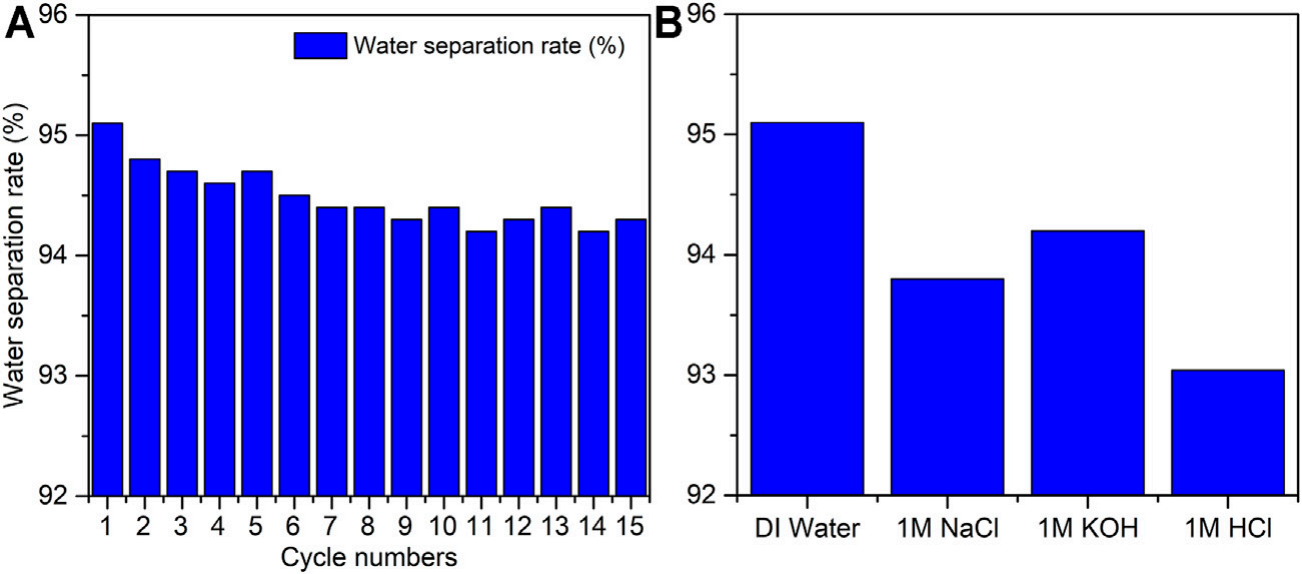
**(A)** Water separation efficiency from the water–oil mixture using the zeolite-coated laser-structured SS-100 meshes during 15 separation cycles. **(B)** Separation efficiencies of the zeolite-coated laser-structured SS-100 mesh in deionized (DI) water, 1 M of NaCl, 1 M of KOH, and 1 M of HCl.

To test the performance of the zeolite-coated laser-structured SS-100 meshes in oil–water separation, experiments were performed as shown in Figure 8 and Supplementary Video S3. In these experiments, the mesh was placed such that the structured and coated surface faces the downward flowing flux. Due to the superhydrophilic surface property, the water passed through the mesh surface in a fraction of seconds while oil was repelled beyond the mesh surface and rejected from passing through. Specifically, the separation of water from the oil–water mixture was carried out through gravitational pull in 18 s with the permeating water flux of 12,738 L·m^−2^·h^−1^ and yielded a separation efficiency greater than 95% with oil intrusion pressure of 1.2 kPa. Figure 9A shows the water separation efficiency for the zeolite-coated laser-structured SS-100 meshes after operation of 15 cycles and reveals that an average efficiency of 94.4% was achieved in the last cycle. These outstanding results demonstrate the durability and applicability of the zeolite-coated laser-structured SS-100 meshes over extended use. This observed stability and reusability of the zeolite-coated laser-structured SS-100 meshes render them as powerful and robust in oil–water separation in industrial processes.

To test the efficiency and durability of the zeolite-coated laser-structured SS-100 meshes under corrosive field conditions, the oil–water separation was conducted in 1 M of NaCl, KOH, and HCl water solutions (Figure 9B). The results reveal that the separation efficiency maintained a value of 94% as compared with the pure water of 95%. Therefore, it can be concluded that there is no significant difference between the performance of these meshes in corrosive media as compared with DI water environment. This could be attributed to the formation of thin films with good chemical and physical stability on the SS-100 mesh substrate, which renders them highly tolerant towards corrosive conditions (Wen et al., 2013; Zhang et al., 2013).

## Conclusion

In this study, NaAlSi_2_O_6_–H_2_O zeolite was grown on femtosecond laser-nanostructured stainless steel substrates. The coated surface was analyzed by XRD, SEM, and EDX and compared with pristine steel meshes, zeolite-coated meshes, and laser-structured meshes, as controls. Detailed contact angle measurements were carried out on pristine steel meshes, zeolite-coated meshes, laser-structured meshes, and zeolite-coated laser-structured-100 mesh specimens. Enhanced superhydrophilic behavior was observed in coated and structured specimens, with the zeolite-coated laser-structured SS-100 meshes exhibiting an average contact angle of 15° along with superior durability over an extended period and repeated use. In addition, the zeolite-coated laser-structured SS-100 meshes were subjected to oil–water separation experiments and revealed augmented effectuation for oil–water separation. In particular, the separation was carried out through gravitational pull in 18 s with the permeating water flux of 12,738 L·m^−2^·h^−1^ and yielded a considerable separating efficiency of 95%.

## Data Availability

The raw data supporting the conclusions of this article will be made available by the authors, without undue reservation.
